# Supplementary material for: Coxsackievirus B3 Infection of Human iPSC Lines and Derived Primary Germ-Layer Cells Regarding Receptor Expression
Source: Int J Mol Sci. 2021 Jan 27;22(3):1220. doi: 10.3390/ijms22031220 (PMC7865966; doi:10.3390/ijms22031220)
Supplement: Supplementary file 1 [file ijms-22-01220-s001.pdf]

International Journal of *Molecular Sciences*

MDPI

Article

Coxsackievirus B3 infection of human iPSC lines and derived primary germ layer cells with regard to receptor expression

Janik Böhnke [1,†], Sandra Pinkert [2,3], Maria Schmidt [4], Hans Binder [4], Nicole Christin Bilz [1], Matthias Jung [5], Uta Reibetanz [6], Antje Beling [2,3], Dan Rujescu [5] and Claudia Claus [1,*]

[1] Institute of Medical Microbiology and Virology, Medical Faculty, University of Leipzig, Johannisallee 30, 04103 Leipzig, Germany; janik.boehnke@rwth-aachen.de (J.B.), christin.emmrich@medizin.uni-leipzig.de (N.C.B.)

[2] Institute of Biochemistry, Berlin Institute of Health (BIH) and Charité -Universitätsmedizin Berlin, corporate member of Freie Universität Berlin, Humboldt-Universität zu Berlin, 10117 Berlin, Germany; sandra.pinkert@charite.de (S.P.), antje.beling@charite.de (A.B.)

[3] DZHK (German Centre for Cardiovascular Research), partner side 10115 Berlin, Germany

[4] Interdisciplinary Center for Bioinformatics, University of Leipzig, 04107 Leipzig, Germany; schmidt@izbi.uni-leipzig.de (M.S.); binder@izbi.uni-leipzig.de (H.B.)

[5] Department of Psychiatry, Psychotherapy, and Psychosomatic Medicine, Martin Luther University Halle Wittenberg, Julius-Kuehn-Strasse 7, 06112 Halle/Saale, Germany; matthias.jung@uk-halle.de (M.J.); dan.rujescu@uk-halle.de (D.R.)

[6] Institute for Medical Physics and Biophysics, Medical Faculty, University of Leipzig, Härtelstrasse 16–18, 04107 Leipzig, Germany; uta.reibetanz@medizin.uni-leipzig.de

* Correspondence: claudia.claus@medizin.uni-leipzig.de; Tel.: ++49 341 97-14321

† Current address Institute for Biomedical Engineering, Department of Cell Biology, RWTH Aachen University, Medical School, Pauwelstrasse 30, 52074 Aachen, Germany

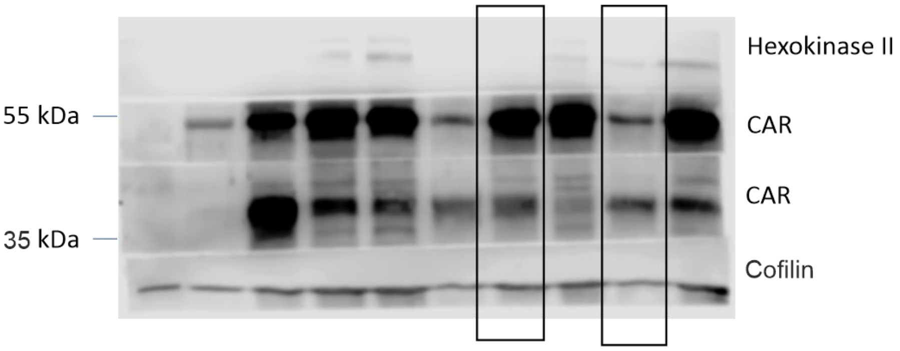

Figure S1. The original Western blot image as depicted in Figure 2C(ii) is given as a reference. The original PVDF Western blotting membrane was horizontally cut in pieces and incubated with the indicated primary antibodies. As CAR antibody clone H-300 (Santa Cruz Biotechnology) was used. The samples shown in Figure 2C(ii) are indicated by a black rectangle.

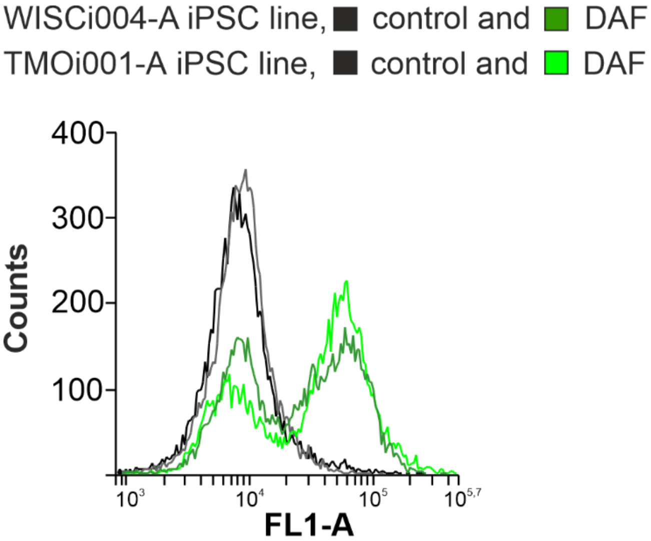

Figure S2. Representative flow cytometric analysis of indicated iPSC lines with anti-DAF and Cy3-conjugated donkey anti-mouse IgG secondary antibody or just secondary antibody (control). The methodology applied confers to the publication by Hübner et al., 2017 [1].

Reference

1. Hübner, D.; Jahn, K.; Pinkert, S.; Böhnke, J.; Jung, M.; Fechner, H.; Rujescu, D.; Liebert, U.G.; Claus, C. Infection of Ipsc Lines with Miscarriage-Associated Coxsackievirus and Measles Virus and Teratogenic Rubella Virus as a Model for Viral Impairment of Early Human Embryogenesis. *ACS Infect. Dis* **2017**, *3*, 886–897.
